# Ectopic expression of S28A-mutated Histone H3 modulates longevity, stress resistance and cardiac function in *Drosophila*

**DOI:** 10.1038/s41598-018-21372-3

**Published:** 2018-02-13

**Authors:** J. P. Joos, A. R. Saadatmand, C. Schnabel, I. Viktorinová, T. Brand, M. Kramer, S. Nattel, D. Dobrev, P. Tomancak, J. Backs, P. Kleinbongard, G. Heusch, K. Lorenz, E. Koch, S. Weber, A. El-Armouche

**Affiliations:** 1 Department of Pharmacology and Toxicology, Medical Faculty, Technische Universität Dresden, Fetscherstraße 74, Dresden, 01307 Germany; 2 Department of Molecular Cardiology and Epigenetics, University of Heidelberg, Heidelberg, Germany; 3 DZHK (German Centre for Cardiovascular Research), partner site Heidelberg/Mannheim, Heidelberg, Germany; 4 Clinical Sensoring and Monitoring, Anesthesiology and Intensive Care Medicine, Faculty of Medicine Carl Gustav Carus, TU Dresden, Fetscherstrasse 74, 01307 Dresden, Germany; 5 Max Planck Insitute of Molecular Biology and Genetics, Pfotenhauerstr. 108, 01307 Dresden, Germany; 6 Comprehensive Heart Failure Center, University of Würzburg, Versbacher Strasse 9, 97078 Wuerzburg, Germany; 7 West German Heart and Vascular Center Essen, University Hospital Essen, Hufelandstrasse 55, 45147 Essen, Germany; 8 Department of Medicine and Research Center, Montreal Heart Institute and Université de Montréal; Departments of Medicine and Pharmacology and Therapeutics, McGill University, Montreal, Canada; 9 Institute of Pharmacology, West German Heart and Vascular Center, Faculty of Medicine, Essen, Germany; 10 Leibniz-Institut für Analytische Wissenschaften – ISAS – e.V., Bunsen-Kirchhoff-Strasse 11, 44139 Dortmund, Germany; 11 Institute of Pharmacology and Toxicology, University of Wuerzburg, Versbacher Strasse 9, 97078 Wuerzburg, Germany

## Abstract

Histone H3 serine 28 (H3S28) phosphorylation and de-repression of polycomb repressive complex (PRC)-mediated gene regulation is linked to stress conditions in mitotic and post-mitotic cells. To better understand the role of H3S28 phosphorylation *in vivo*, we studied a *Drosophila* strain with ectopic expression of constitutively-activated H3S28A, which prevents PRC2 binding at H3S28, thus mimicking H3S28 phosphorylation. H3S28A mutants showed prolonged life span and improved resistance against starvation and paraquat-induced oxidative stress. Morphological and functional analysis of heart tubes revealed smaller luminal areas and thicker walls accompanied by moderately improved cardiac function after acute stress induction. Whole-exome deep gene-sequencing from isolated heart tubes revealed phenotype-corresponding changes in longevity-promoting and myotropic genes. We also found changes in genes controlling mitochondrial biogenesis and respiration. Analysis of mitochondrial respiration from whole flies revealed improved efficacy of ATP production with reduced electron transport-chain activity. Finally, we analyzed posttranslational modification of H3S28 in an experimental heart failure model and observed increased H3S28 phosphorylation levels in HF hearts. Our data establish a critical role of H3S28 phosphorylation *in vivo* for life span, stress resistance, cardiac and mitochondrial function in *Drosophila*. These findings may pave the way for H3S28 phosphorylation as a putative target to treat stress-related disorders such as heart failure.

## Introduction

Phosphorylation of histone H3 (H3) is an important downstream signalling event that couples signal transduction pathways, gene regulation and biological processes^1,2^. Transient and rapid phosphorylation of H3 at serine 10 and serine 28 is evolutionarily conserved^3,4^. Besides the well-studied role of H3 phosphorylation during mitosis, recent studies in neurons and cardiomyocytes further show a stress-induced (e.g. by phorbol esters or calcium overload) increase of H3, or in particular of H3S28 phosphorylation^5,6^. H3S28 phosphorylation levels are fine-tuned by context-dependent specific kinases and phosphatases, e.g. MSK1/2, aurora-B and protein phosphatase 1^7–9^. *In vitro* and cell-based studies indicated that increased H3S28 phosphorylation can induce H3K27 acetylation and thereby antagonize polycomb-mediated silencing by diminishing H3K27 methylation^5,7,10^, a potent and widespread transcriptional repression indicator^11^. The polycomb repressive complexes (PRC 1 and 2) coordinate this process^12^. In order to understand the functional outcome of mild to severe impairments in PRC constitution, different *Drosophila* mutant strains were analysed in terms of longevity, stress resistance and oxidative capacity^13–17^. These studies suggested that mild changes in PRC function increase longevity and oxidative stress resistance, while stronger changes are detrimental^18^. The essential upstream function of H3S28 within PRC (de)-repression was recently shown in a new *Drosophila* mutant strain carrying a serine-to-alanine (H3S28A) replacement^18^. Mosaic replacement of H3S28A mimicked H3S28 phosphorylation in the targeted cells without any effects on mitosis but with strongly decreased H3K27 methylation-levels. Gross phenotypic analysis showed homeotic transformations like antenna-to-leg transformations in adult tissues, underlining the importance of H3S28 in PRC-orchestrated transcriptional processes related to maintenance of cell identity. These findings raise the possibility that a more moderate modulation of H3S28-mediated PRC axis de-repression might also affect yet unexamined key biological processes like life span, organ integrity and stress resistance. Moreover, there is emerging evidence that increased H3S28 phosphorylation correlates with chronic stress-related diseases states (e.g. atherosclerosis and stroke) in mammalian post-mitotic cells^5,6^. Here, we examined a *Drosophila* strain with ectopic expression of a S28A-mutated histone H3 on wild-type background to mimic mild PRC de-repression in the presence and absence of stress conditions. We did not observe homeotic transformations in the transgenic *Drosophila* strains but found significant changes in heart morphology and function that were paralleled by longer lifespan, greater stress resistance and better mitochondrial function.

Due to the complexity of genomic histone H3 coding sequences in vertebrate models, the best current state-of-the-art animal model to study the impact of aberrant H3S28 phosphorylation is a transgenic *Drosophila* melanogaster model with an H3S28A replacement. This mutant reduces the ability of PRC2 to methylate H3K27 on nucleosomal substrates, thus mimicking increased H3S28 phosphorylation and subsequent H3K27 acetylation^19^. In order to circumvent homeotic transformation and thereby exclude influence of developmental organ defects on basal and stress-induced phenotypes of adult flies, we studied the H3S28A mutation on a wild-type background^18^. This led to an estimated ratio of approximately 100:6 of wild-type to mutant H3S28 alleles and avoided overt signs of homeotic transformation (data not shown). Transgenic H3S28S flies with intact PRC2-mediated downstream signalling were used as a control. Genotyping, as well as transcriptional analysis, showed that both mutant strains carried the H3S28A or H3S28S insertions as intended (Suppl. Figure 1).

## Results

### H3S28A Mutants Show Increased Longevity and Improved Stress Resistance *In Vivo*

**Figure 1:**
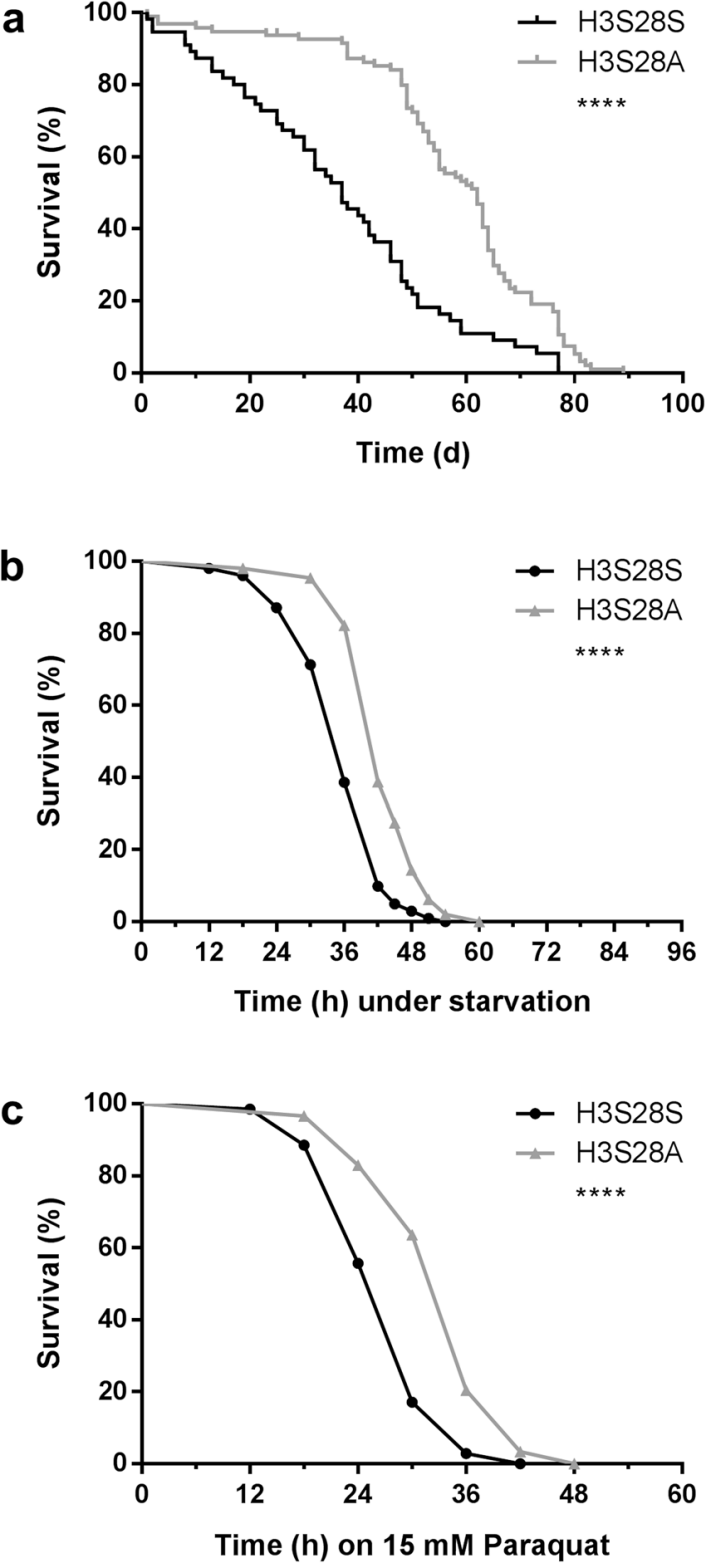
H3S28A mutants exhibit a prolonged median lifespan and an increased resistance to starvation and oxidative stress. (**a**) Median lifespan of H3S28A mutants is increased about 68% (n_H3S28S_ = 56, n_H3S28A_ = 96). (**b**) Median survival during acute starvation is increased about 16% in H3S28A mutants (n_H3S28S_ = 101, n_H3S28A_ = 152; 7–10 days old). (**c**) Median survival during acute exposure to 15 mM paraquat is increased about 20% in H3S28A mutants (n_H3S28S_ = 70, n_H3S28A_ = 88; 7–10 days old). Each curve represents the average of at least 3 separate experiments.

To determine whether transgenic overexpression of H3S28A modulates life span and stress resistance, we first examined male flies, to avoid experimental confounding due e.g. nutrition influence that is elevated in Drosophila females to produce eggs. We found a nearly 70% increase in median life span of H3S28A mutants under basal conditions (Fig. 1a), an increased resistance against food withdrawal (Fig. 1b), and a greater resistance against oxidative stress conditions in the paraquat feeding model (Fig. 1c). In addition, we observed identical results in female flies under basal and stress conditions (Suppl. Figure 2a–c). These data indicate a critical role for H3S28 phosphorylation on life span and stress resistance *in vivo*.

### H3S28A Mutants Have Smaller Heart Lumina, Higher Heart Rate and a better Acute Stress Response

**Figure 2:**
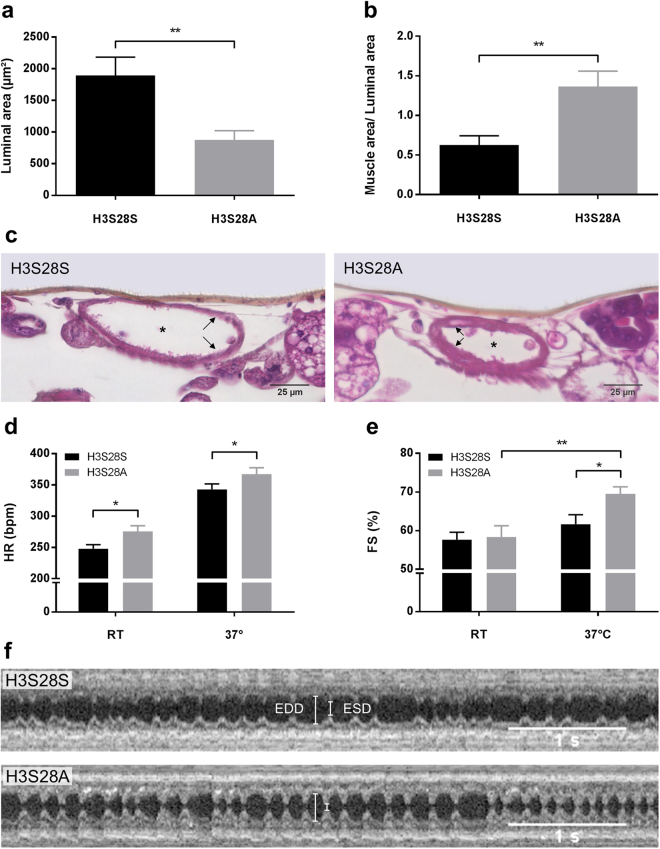
Histomorphological and functional analysis of H3S28S and H3S28A heart tubes. Quantification of the (**a**) luminal area and (**b**) heart muscle area over the entire cross section normalized to the luminal area (n_H3S28S, H3S28A_ = 11, 7 days old). (**c**) Representative cross sections of H3S28S and H3S28A heart tubes at the level of the conical chamber. Arrows mark the heart wall; asterisks mark the heart lumen. (**d**) Heart rate and (**e**) fractional shortening (FS) as a measure for the cardiac contractile function. All measurements were performed at room temperature (RT) and following thermal stimulation (37 °C) for at least five minutes (n_H3S28S_ = 22, n_H3S28A_ = 24; 2 days old). (**f**) Exemplary M-mode OCT registrations from H3S28S and H3S28A flies following thermal stimulation. EDD: end-diastolic diameter; ESD: end-systolic diameter.

Aging in *Drosophila* is associated with decreased heart function and vulnerability to heart failure^20,21^. Thus, we compared cardiac morphological and functional features of the two fly strains. H&E staining in histological sections of H3S28S vs. H3S28A heart tubes (Fig. 2c) revealed smaller luminal areas in H3S28A vs. H3S28S flies (H3S28S vs. H3S28A: 1895 ± 288 vs. 877 ± 145 µm²) (Fig. 2a). Accordingly, since absolute heart muscle area was identical between H3S28A and H3S28S flies, the ratio of muscle area (and cardiac wall thickness) compared to luminal area was greater in H3S28A flies (H3S28S vs. H3S28A muscle area/luminal area: 0.63 ± 0.12 vs. 1.37 ± 0.19) (Fig. 2b and Suppl. Figure 8). Next, we examined functional cardiac parameters using non-invasive optical coherence tomography (OCT)^22^ and subjected the flies to a well-established cardiac stress model by increasing the ambient temperature (to 37 °C) 5 minutes before and during OCT measurement^23^. H3S28A mutant flies showed higher heart rates both at room temperature and at 37 °C (H3S28S vs. H3S28A RT: 248 ± 7 vs. 276 ± 9 bpm; 37 °C: 343 ± 9 vs. 367 ± 10 bpm) (Fig. 2d). Notably, the relative response in heart rate did not differ. Both strains showed a reduced arrhythmicity index at 37 °C, while there was no difference in arrhythmic events in response to temperature stress (Suppl. Figure 3). While both strains showed comparable fractional shortening under basal conditions (H3S28S vs. H3S28A: 57.7 ± 1.9 vs. 58.4 ± 2.9%), only H3S28A mutants showed increased contractility upon temperature stress (H3S28S vs. H3S28A: 62 ± 2 vs. 70 ± 2%) (Fig. 2e).

### Whole-Exome Deep Gene Sequencing Reveals Differential Expression of Longevity-Promoting and Mitochondrial Biogenesis and Respiration Genes in H3S28A Mutants

**Figure 3:**
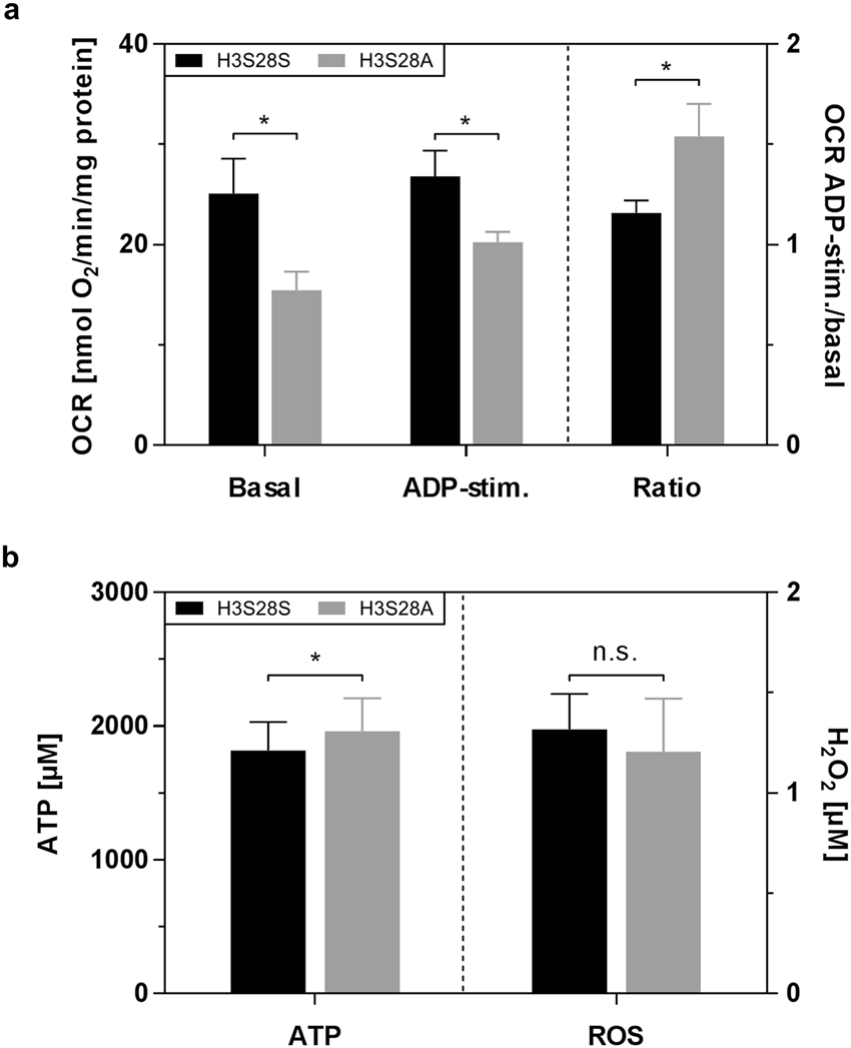
Mitochondria of H3S28A mutants show a generally reduced oxygen consumption with increased release of ATP and unchanged H_2_O_2_ production. (**a**) Oxygen consumption rate under basal complex-I-respiration and after stimulation with 1 mM ADP (left) and ratio of both states (right). Results are normalized to maximal uncoupled respiration via 30 mM FCCP (n_H3S28S_, n_H3S28A_ = 11 samples à 20 flies, 7–10 days old). (**b**) Production of ATP (left) and ROS (right) immediately after quantification of ADP-stimulated respiration (n_H3S28S_, n_H3S28A_ = 7 [ATP]/8 [ROS] samples à 20 flies, 7–10 days old).

Posttranslational modifications on position K27 and S28 of the histone H3 tail lead to changes in the transcriptional landscape of different cell types. To decipher the genetic networks involved in the H3S28A mutant, we conducted whole-genome RNA sequencing analysis from isolated heart tubes of adult flies. We found 737 differentially expressed genes, of which 391 were significantly up- and 346 significantly down-regulated (Suppl. Figure 4, Suppl. data set 1 [orange color]). Partial de-repression of polycomb signalling was confirmed by up-regulation of polycomb-repressed genes, such as Cyp6a8 and Cbp53E (Suppl. Table 1). We also found a substantial number of genes with confirmed or predicted function in myocardial contractility or ageing/longevity (Suppl. Figure 5), consistent with the observed increase in life span and fractional shortening in the mutant flies. Moreover, we found a significantly over-represented subset of genes with predicted localization and/or function in mitochondria by unbiased GO enrichment analysis (http://www.geneontology.org/page/go-enrichment-analysis; Suppl. Figure 7). Previous studies in flies as well as in higher vertebrate animal models suggested that proper mitochondrial function is crucial to resist paraquat-induced oxidative stress. We studied respiration under basal and ADP-stimulated conditions, as well as ROS and ATP production, finding lower basal as well as stimulated mitochondrial respiration in mitochondria of whole H3S28A flies (Fig. 3a). Interestingly, reduced mitochondrial respiration was paralleled by increased ATP production, implying increased efficiency of the respiratory chain (Fig. 3b left). In contrast, we did not find any differences in ROS production between fly strains (Fig. 3b right). Together these data indicate better mitochondrial electron transport chain efficiency with unchanged ROS production in the H3S28A mutant flies.

### Increased H3S28 Phosphorylation and H3K27 Acetylation in Atria of Failing Hearts

**Figure 4:**
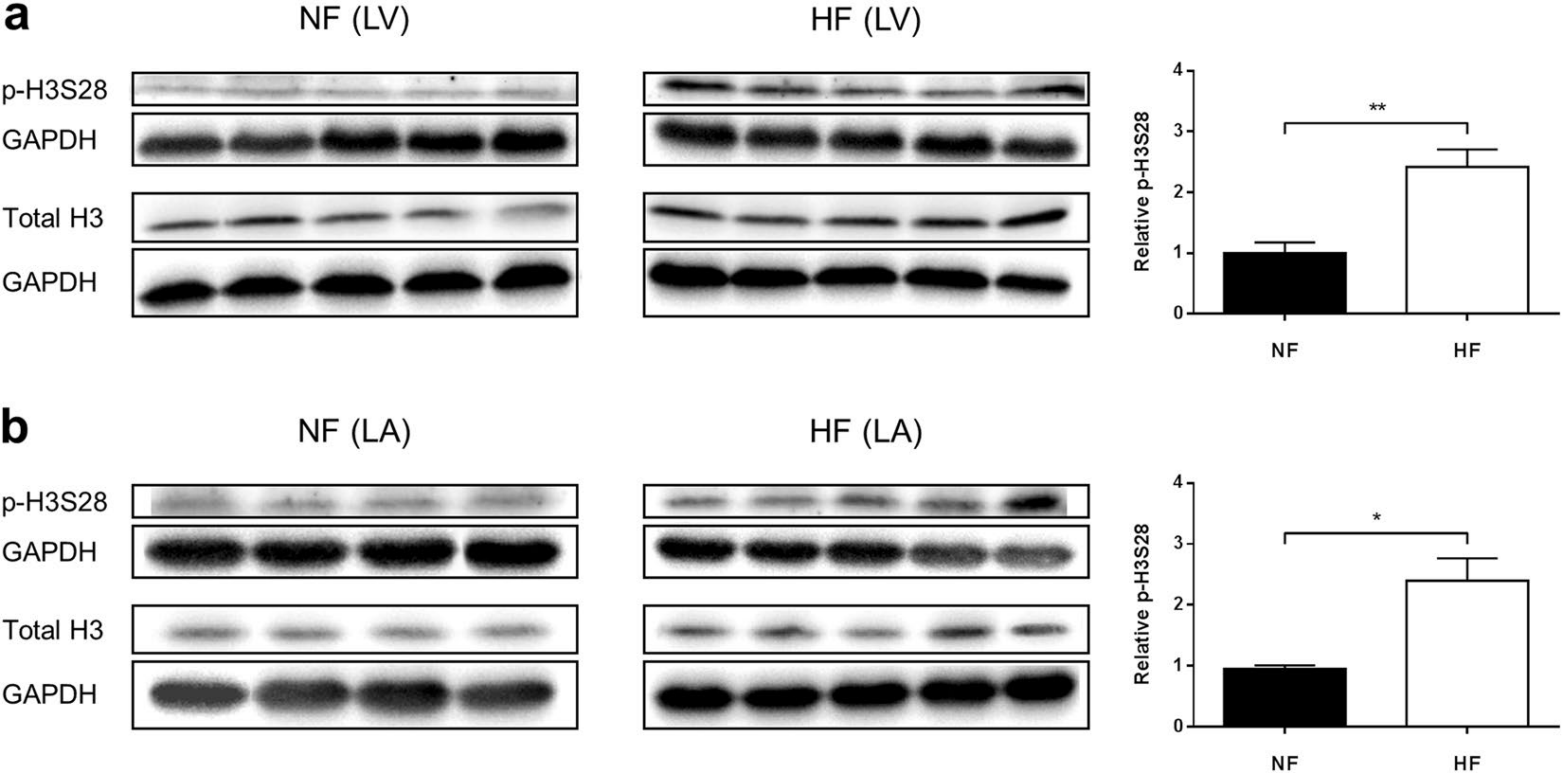
Western Blot analysis of p-H3S28 levels (normalized to total histone H3) in left ventricular (LV; **a**) and left atrial (LA; **b**) heart tissue of dogs with pacing-induced heart failure (HF) and respective non-failing controls (NF). All samples were normalized to GAPDH. Presented signals from NF and HF tissues were cropped from one continuous Western blot which is displayed as Suppl. Figure 9 (indicated by boxes).

*Drosophila* is frequently used as a disease model for cardiomyopathies, including heart failure^23,24^. However, because of well-known differences between the cardiac physiology of flies and mammals the translation from *Drosophila* to humans is challenging. To assess the potential relevance of H3S28 phosphorylation in a clinically relevant disease model we focused on heart failure (HF), commonly associated with acute and chronic cardiac stress conditions as well as preceding cardiac hypertrophy. Indeed, in a pacing-induced heart failure model in dogs^25,26^ we found increased H3S28 phosphorylation in failing ventricular and atrial myocardium (Fig. 4a–b and Suppl. Figure 9). We also found greater H3K27 acetylation in atria of failing myocardium but not in ventricles (Suppl. Figure 7a–b), while H3K27 di-methylation was unchanged for both regions (data not shown). As recent findings suggested that H3S10 phosphorylation might be altered in cardiac cells under stress conditions^27,28^ we also studied H3S10 phosphorylation in ventricles and atria of the same samples. Interestingly, we did not observe differences in H3S10 phosphorylation in ventricles of HF vs. NF hearts, whereas H3S10 phosphorylation levels were modestly but significantly increased in the atria of HF hearts (Suppl. Figure 7c–d). Thus, H3S28 phosphorylation is increased in stressed mammalian hearts and might play an adaptive role in HF.

## Discussion

The results presented here point to a potential role for H3S28 phosphorylation in controlling longevity, stress resistance and cardiac function *in vivo* in *Drosophila*.

The moderate de-repression of PRC2 function in the H3S28A mutant *Drosophila* strain led to a nearly doubled median life span under basal living conditions. This is consistent with other fly strains showing moderate de-repression of PRC2 signalling, e.g. by heterozygous mutation of the H3K27 methyltransferase E(z) or the Suppressor of zeste 12 (Su(z)12)^13,15,16^. It is important to note, however, that polycomb silencing must not be seen as a classic on-off switch but rather as a “governor” to selectively modulate target gene expression^29–31^. This theory is strengthened by studies showing lethal effects in different animal models in which polycomb signalling was strongly modulated^32,33^. We also demonstrate greater resistance against starvation and oxidative stress in the H3S28A mutant flies. In fact, starvation was shown to be a strong producer of oxidative stress in *Drosophila*, arguing for a common mechanism of stress protection in both models of chronic stress used in this study^34–36^.

We demonstrate regulation of at least seven genes with predicted or experimentally validated influence on life span and at least six genes with potential functions in the contractile apparatus of cardiomyocytes (Suppl. Figure 5). The latter correlates well with results from a transverse aortic construction mouse model of cardiac hypertrophy showing increased H3K27-acetylation at transcription start sites of genes which affect cardiac contraction^37^. Most of them have not yet been studied in the context of cardiac physiology or cardiac disease models *in vivo*. Analysis of the differentially expressed genes showed that only a few, e.g. Cyp6a8 and Cbp53E, out of the hundreds of known polycomb target genes in *Drosophila* were upregulated in H3S28A compared to H3S28S hearts. (Suppl. Table 1) This again is consistent with moderate effects on the PRC2 silencing machinery. In addition, we propose a previously unknown role for H3S28-dependent regulation of mitochondrial gene expression, as shown by unbiased GO enrichment analysis (http://www.geneontology.org/page/go-enrichment-analysis, Suppl. Figure 6). The impact on mitochondrial gene expression in heart tubes is underlined by our data showing greater ATP production under reduced electron transport chain activity in whole flies. These data suggest a favourable cardiac phenotype in H3S28A that may exhibit features of a better response to stress in mammals and indicate transcriptional regulation of metabolic genes as a novel feature of PRC-mediated functional responses in adult tissues of *Drosophila*^38,39^.

We demonstrate smaller heart luminal areas accompanied by relatively thicker heart walls and moderately higher heart rates in H3S28A flies. As proper heart function is a critical factor of basal lifespan as well as greater resistance against starvation and oxidative stress, it was not surprising that the H3S28A heart tubes showed a favourable cardiac phenotype. In contrast, cardiac tube hypertrophy in transgenic or environmentally challenged *Drosophila* models lead to either pathological manifestations of cardiac hypertrophy with a concomitant decrease in heart function^40,41^ or a clear transition into systolic or diastolic HF and decreased life span^42–44^. The mildly improved cardiac performance after acute temperature stress indicates better cardiac stress responses, which might translate into cardioprotection under conditions like cardiac surgery or ischemia/reperfusion. This is of particular importance because many studies have tried to reveal (epi-)genetic checkpoints which control the transition from cardiac hypertrophy to heart failure^42,45–50^. Identifying such checkpoints would be extremely helpful to identify novel pharmacological targets to delay the progression of cardiac hypertrophy towards heart failure.

Interestingly, we found greater H3S10 phosphorylation exclusively in atria of chronically stressed dog hearts (Suppl. Figure 7c–d). This finding might be interesting in the light of previous studies showing greater H3S10 phosphorylation levels of murine and human cardiac tissues under chronic stress conditions^27,28^. While regulation of H3S28 phosphorylation has been detected in mitotic as well as in post-mitotic cells, H3S10 phosphorylation was primarily shown in mitotic cells^1,2^. Thus, increased levels of H3S10 phosphorylation in cardiac tissues might be related to mitotic events in non-cardiomyocyte cells like fibroblasts or endothelial cells^19,51–53^. Interestingly, we also observed a correlation between H3S28 phosphorylation and H3K27 acetylation in the atria, which is again in line with former studies of a transverse aortic construction mouse model of cardiac hypertrophy^37^. This fits well to previous *in vitro* studies showing a causative relationship between H3S28 phosphorylation and subsequent H3K27 acetylation, while methylation of H3K27 was inhibited. This model is called the “phospho-methyl-acetyl switch” and might be the underlying cause for our findings of modulated H3S28 phosphorylation and H3K27 acetylation levels^54^. In addition, increased H3K27 acetylation may also reflect an activation of cardiac fibroblasts in the atria of chronically stressed dog hearts which is consistent with other studies showing increased H3K27 acetylation as a marker of fibroblast activation^53,55^.

Our study has several limitations. For instance, our data on mitochondrial respiration obtained in whole flies might not represent the situation in the heart. Therefore, it would be of great interest to confirm the mitochondrial respiration data obtained in whole flies at the level of isolated heart tubes, which would require the development of novel techniques with improved sensitivity. In addition, we cannot rule out additional (repressing) effects of ectopic H3S28A overexpression on wildtype Histone H3 levels, which may contribute to the pleiotropic responses observed in our drosophila model. Finally, we validated our findings obtained in flies in dogs with clinical manifestation of HF. It will be important to study the phosphorylation state of H3S10 and H3S28 in animal models with compensated hypertrophy but no signs of clinical HF.

In conclusion, our data establish an important modulatory role of ectopic H3S28A expression and thus point to a potential role of H3S28 phosphorylation *in vivo* for life span, stress resistance and cardiac stress responses. Furthermore, to our knowledge we present the first genetic *Drosophila* model with an improved cardiac phenotype. The next experimental step should include directed mutagenesis of H3S28 in a larger animal model, however sophisticated gene targeting strategies as CRISPR/Cas9 will be needed to address this issue due to the complex and widespread organization of the Histone H3 cluster in mammalian genomes. Nevertheless, our data offer a starting point to find potential avenues towards drug development to therapeutically modulate stress-related human diseases such as heart failure.

## Material and Methods

### Drosophila stocks

*D. melanogaster* H3S28A (*y, w, hsp70-flp122; P(Ubi-GFP.D)33, P(Ubi-GFP.D)38, FRT40A; 6xHisGU*^*H3S28A*^) and control strain H3S28S (*y, w, hsp70-flp122; P(Ubi-GFP.D)33, P(Ubi-GFP.D)38, FRT40A; 6xHisGU*) were grown on standard medium at RT. The generation of the transgenic strains was described in detail before^18,56^ (https://tel.archives-ouvertes.fr/tel-01199169).

### Life span assays

Few hours after eclosion, 20–25 flies of the same sex were placed into vials containing standard medium. Flies were kept at room temperature (RT) and transferred to fresh food without anesthesia at least every five days. Dead flies were scored on a daily basis until all flies were dead. Each experiment was repeated at least three times and results were pooled to generate survivorship curves.

### Starvation Resistance Assays

Starvation resistance assays were performed similar as described before^13^: 7- to 10-day-old flies of the same sex were placed into vials (20–70 flies/vial) containing 4 ml of 1.3% low-melt agar with water. Flies were kept at RT for the duration of the assay. Dead flies were scored four times a day until all flies were dead. Each experiment was repeated three times and results were pooled to generate survivorship curves.

### Oxidative Stress Resistance Assays

Oxidative stress resistance assays were performed similar as described before^13^: 7- to 10-day-old flies of the same sex were placed in vials without food or water for 4 h at RT, then placed in vials (20–50 flies/vial) containing 4 ml of media [5% sucrose, 1.3% low-melt agar, and 15 mM paraquat (Sigma-Aldrich)]. Paraquat was added when media temperature reached 45 °C to prevent heat inactivation. Assays were conducted at RT, dead flies were scored four times a day until all flies were dead. Each experiment was repeated three times and results were pooled to generate survivorship curves.

### OCT measurement of heart rate and fractional shortening

Cardiac function of 2-day-old male flies was measured using a custom built OCT system (Clinical Sensoring and Monitoring, Technische Universitaet Dresden,^57^). A detailed description of the fly preparation, measurement and analysis of beat rate, arrhythmicity index as well as fractional shortening can be found in the supplementary Material and Methods section.

### Histological analysis

Histological analysis was conducted similar as described before^40^: 7-day-old male flies were collected and fixed in Telly’s fixation buffer (60% ethanol, 3.33% formalin, 4% glacial acetic acid) at 4 °C. The cardiac chamber sizes in flies from this fixation step are similar to the end-diastolic diameter in alive adult flies as assessed using OCT^58^. Specimens were then dehydrated in sequential gradients of ethanol and washed twice with Xylene before immersion in liquid paraffin. After solidification, paraffin blocks were sectioned serially at 6 μm thickness in transverse orientation. Sections were rehydrated and stained with hematoxylin and eosin. Established criteria were used to control for the position of the heart chamber among different flies^59^. Measurements were analyzed in two serial sections using a Keyence BZ-X710 microscope (magnification of 40×) and the appropriate BZ-X Analyzer Software. Heart wall area was determined by calculating the difference between the areas enclosed by the contours around the entire heart and around the lumen (Suppl. Figure 8). To ensure better comparability, the heart wall area was normalized to the luminal area.

### Differential gene expression analysis

A detailed description of the differential gene expression analysis can be found in the supplementary Material and Methods section. In brief, cardiac tubes of 7-day-old male flies were dissected and exposed according to Vogler and Ocorr^60^. RNA was extracted and the integrity was evaluated using an RNA 6000 Pico assay on an Agilent 2000 Bioanalyzer according to the manufacturer’s instructions. RNA sequencing and statistical analysis were carried out as described before^61^ and as detailed in the supplementary Material and Methods section.

### Mitochondrial function

A detailed description of the mitochondrial function analysis can be found in the supplementary Material and Methods section and as described before^62^. In brief, mitochondria were isolated from 7- to 10-day-old male flies and respiration was measured using a Clark-type electrode (Strathkelvin) at 37 °C. For the measurement of complex I respiration, ADP was added and ADP-stimulated respiration was measured for 3 min^63,64^. Maximal uncoupled respiration was measured by adding 30 nM FCCP (Carbonyl cyanide-4-(trifluoromethoxy)phenylhydrazone) and subsequent analysis for 30 s. Respiration was calculated as nmol O_2_/min/mg protein using the Stratkelvin analysis software. ATP production was determined from mitochondrial supernatant and compared with ATP standards using a 96-well white plate and a Cary Eclipse spectrophotometer (Varian) at 560 nm emission wavelength. Mitochondrial ROS production was determined using the Amplex Red Hydrogen Peroxide Assay (Life Technologies).

### Pacing-induced heart failure model in dogs, Cardiac tissue preparation, SDS-Gel electrophoresis and immunoblotting

All animal care and handling procedures followed the Guidelines of the Canadian Council for Animal Care. Ventricular pacing-induced canine HF was produced with well-established procedures^26^. Canine cardiac tissue preparation was performed as described before^65^. In brief, the tissue was rapidly frozen by nitrogen, pulverized and lysed in lysis buffer (30 mmol/L Tris/HCl pH 8.8, 5 mmol/L EDTA, 30 mmol/L NaF and 3% SDS) supplemented with protease inhibitor cocktail and phosphatase inhibitor cocktail (Roche Diagnostics). The protein samples were separated by weight using SDS-polyacrylamide gel electrophoresis and subsequently transferred to nitrocellulose membranes. Membranes were blocked with 5% non-fat milk (AppliChem) for 1 h and afterwards incubated with the respective primary antibody overnight at 4 °C. After repeated washing steps and incubation with appropriate secondary antibodies for 1 h, chemiluminescence was detected using a Fusion FX imaging system (Vilber Lourmat) and quantified with Fusion-Capt Advance (Vilber Lourmat) software. Antibodies were from Santa Cruz (Gapdh, sc-365062), Cell Signalling (Histone 3, #4499; p-H3S28, #9713) or Abcam (ac-H3K27, ab4729).

### Statistical analysis

Statistical analysis was performed using Graph Pad Prism 6 (Graph Pad) using the following tests:

- HR and FS: Two-way ANOVA with Bonferroni post-hoc test
- Heart muscle- and luminal area: Unpaired t test with Welch’s correction
- Survival and stress: Log-rank test
- Mitochondrial function: Unpaired t test with Welch’s correction
- Differential gene expression: See supplementary Material and Methods section
- All data are presented as means ± SEM, *p ≤ 0.05, **p ≤ 0.01, ***p ≤ 0.001, ****p ≤ 0.0001.

## Electronic supplementary material

Supplementary information

Dataset 1
