# Supplementary material for: Ectopic expression of S28A-mutated Histone H3 modulates longevity, stress resistance and cardiac function in Drosophila
Source: Sci Rep. 2018 Feb 13;8:2940. doi: 10.1038/s41598-018-21372-3 (PMC5811592; doi:10.1038/s41598-018-21372-3)
Supplement: Supplementary file 1 — Supplementary information [file 41598_2018_21372_MOESM1_ESM.pdf]

Supporting Information

Ectopic expression of S28A-mutated Histone H3 modulates longevity, stress resistance and cardiac function in *Drosophila*

J. Joos[1]*, A.R. Saadatmand[2]*, C. Schnabel[3], I. Viktorinová[4], T. Brand[5,6], M. Kramer[1], S. Nattel[6,7], D. Dobrev[8], P. Tomancak[4], J. Backs[2], P. Kleinbongard[6], G. Heusch[6], K. Lorenz[5,6,9,10], E. Koch[3], S. Weber[1]#, A. El-Armouche[1]#

Supplementary Figures and Legends

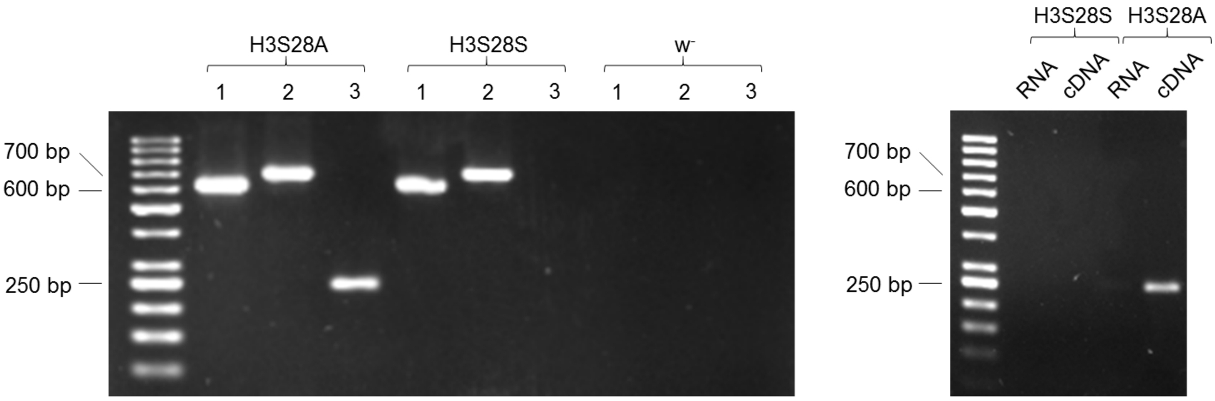

Suppl. Fig. 1. PCR detection of H3S28S or H3S28A transgene insertion. (Left) Verification on DNA level. Bands 1 (~ 600 bp) and 2 (~ 650 bp) show the insertion of the three Histone gene units on chromosome 3L (locus 86E) and 3R (lous 86 Fb). Band 3 (250 bp) confirms the H3S28A mutation within the transgenes. w^- flies were run as controls. (Right) Verification of the expression of the mutated Histone H3 on RNA level. Since the transgenes do not contain any introns, samples without reverse transcription were run as controls to prevent contamination with gDNA.

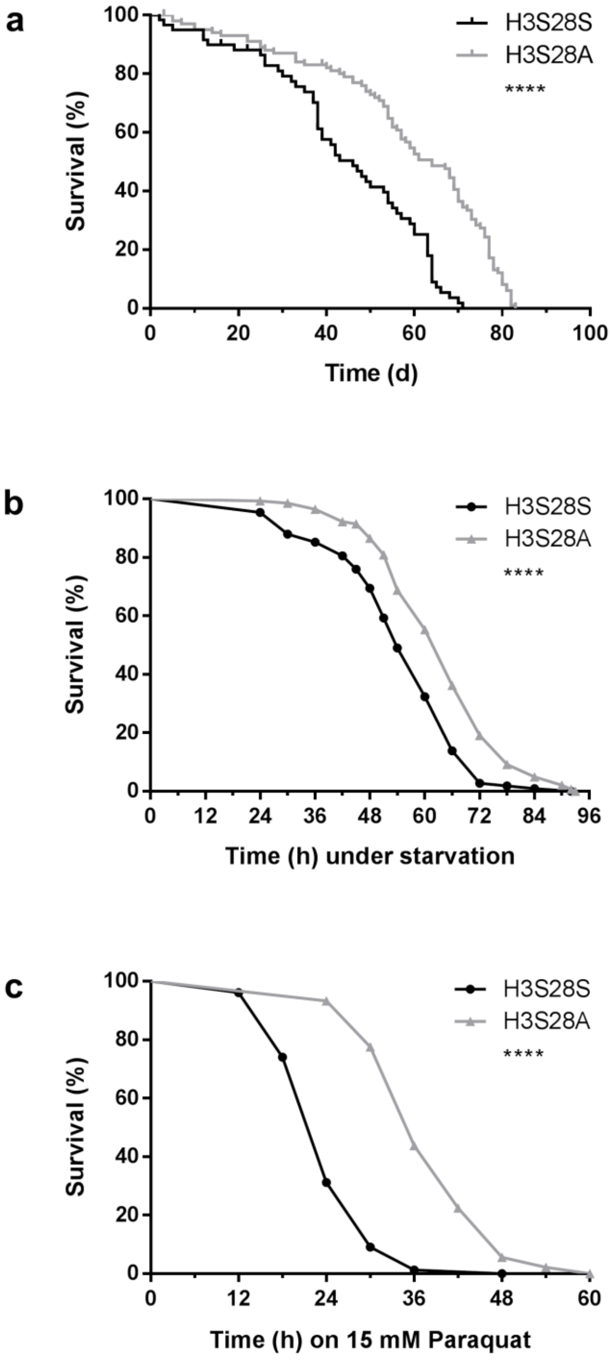

Suppl. Fig. 2. Female H3S28A mutants exhibit a prolonged median lifespan and an increased resistance to starvation and oxidative stress. (**a**) Median lifespan of female H3S28A mutants is increased about 39% (n_{H3S28S}=60, n_{H3S28A}=102). (**b**) Median survival during acute starvation is increased about 22% in female H3S28A mutants (n_{H3S28S}=108, n_{H3S28A}=141; 7–10 days old). (**c**) Median survival during acute exposure to 15 mM paraquat is increased about 50% in female H3S28A mutants (n_{H3S28S}=77, n_{H3S28A}=89; 7–10 days old). Each curve represents the average of at least 3 separate experiments.

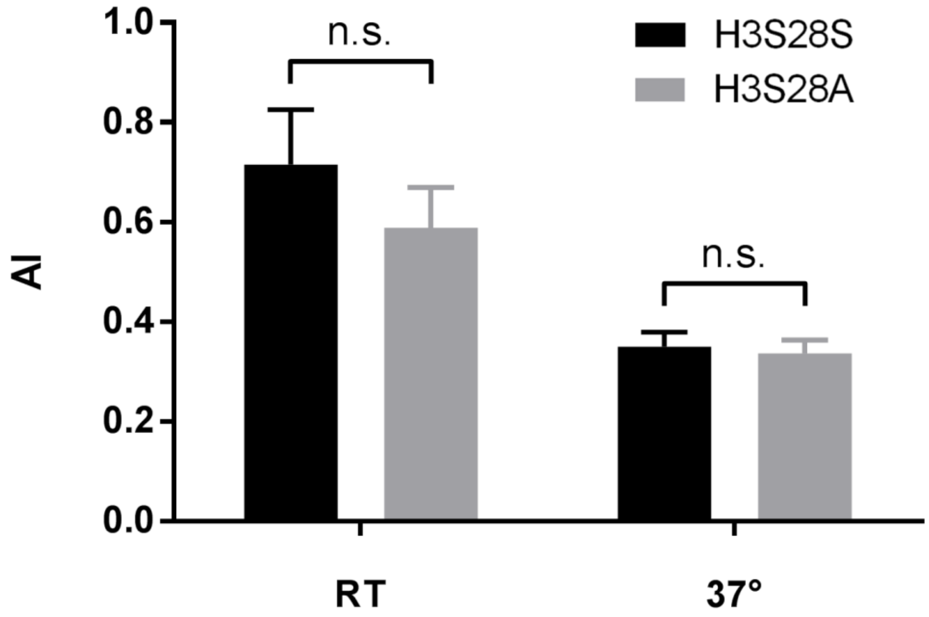

Suppl. Fig. 3: Unchanged arrythmicity index in H3S28A mutants. The AI is defined as the standard deviation of the heart periods normalized to the median heart period. It increases with decreasing rhythmicity. All measurements were performed at room temperature (RT) and following thermal stimulation (37 °C) for at least five minutes (n_{H3S28S}=22, n_{H3S28A}=24; 2 days old males).

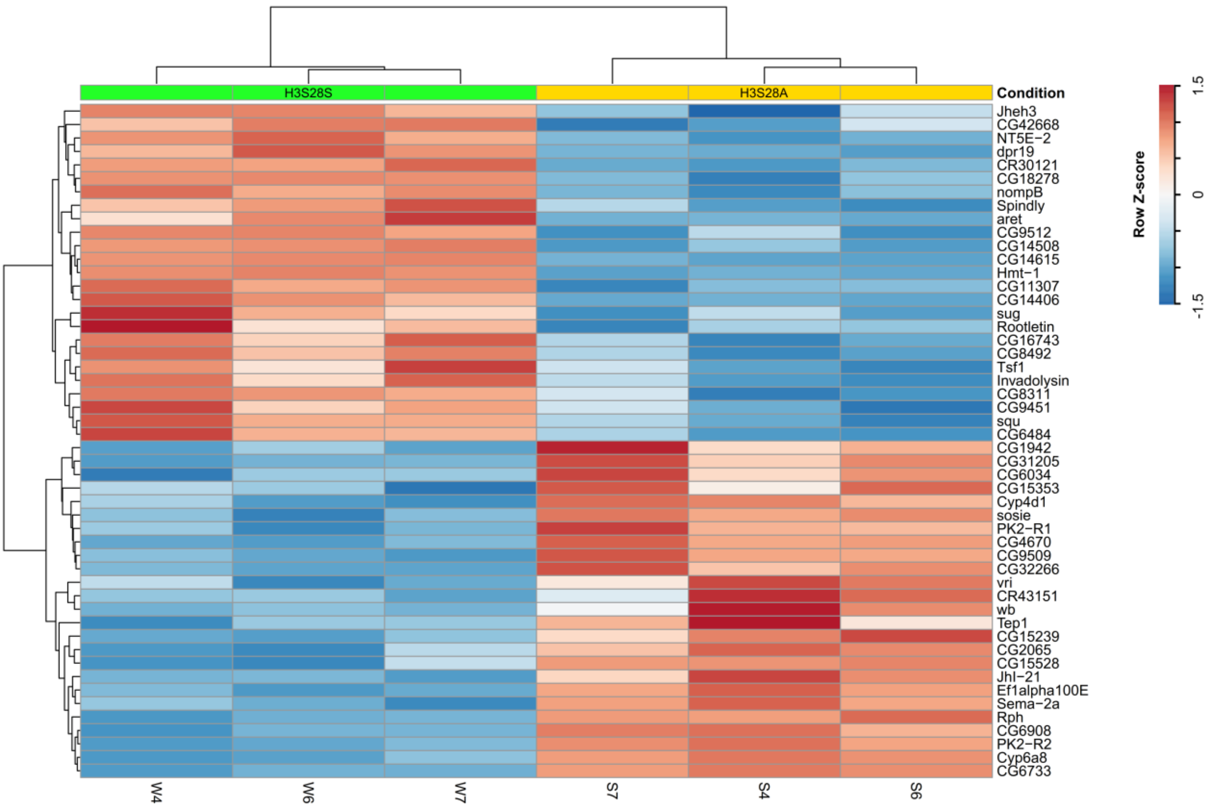

Suppl. Fig. 4: Heatmap of the 25 most up- and down-regulated genes. The color code represents the row z-score, where a value over 0 (bright to dark red) corresponds to an increased and a value below 0 (bright to dark blue) corresponds to a decreased expression compared to the arithmetic mean over all samples. The arrangement of the samples (columns) and genes (rows) corresponds to the hierarchical clusters (see dendrograms above an left) ($n_{H3S28S,H3S28A}$ = 3 samples à 15 heart tubes; 7 days old males).

Gene symbol and –name	FC	Function of the gene product	Reference
**Survival/ aging/ stress resistance**			
**CG9509**	5,86	– Choline dehydrogenase (?)<br>– Adaption to temperature or humidity<br>– Metabolic detoxification	Glaser-Schmitt et al., 2013
**CG15239**	4,26	– Unknown function<br>– Longevity-promoting	Chittaranjan et al., 2009
**Ef1alpha100E (Elongation factor 1alpha100E)**	4,17	– Longevity-promoting	Shepherd et al., 1989
**CG4670**	2,95	– Flavin-linked sulfhydryl oxidase activity (?)<br>– Carbohydrate metabolism, electron transport<br>– Longevity-promoting	Curtis et al., 2007
**Obp99b (Odorant-binding protein 99b)**	1,71	– Humoral signaling factor (?)<br>– Longevity-promoting	Alic et al., 2014
**GstE3 (Glutathione S transferase E3)**	1,71	– Detoxification	Zhikrevetskaya et al., 2015
**l(2)efl (lethal (2) essential for life)**	1,58	– Small heat shock protein<br>– Overexpression extends lifespan and resistance to oxidative stress	Tanguay und Morrow, 2008
**Myotropic/ heart rate**			
**vri (vrille)**	2,31	– bZIP transcription factor<br>– Cardiac survival factor (anti-aging and –senescent)<br>– E4BP4 (human homolog) overexpression in human heart failure	Weng et al., 2010; Monnier et al., 2012
**wb (wing blister)**	2,17	– Laminin α1,2 chain<br>– Mutation leads to defective heart development<br>– Loss of human homolog (merosin) is involved in muscular dystrophies including heart failure	Martin et al., 1999; Finsterer et al., 2010
**TpnC47D (Troponin C at 47D)**	1,78	– Calcium-binding regulatory protein in striated muscle<br>– Increased expression during embryogenesis	
**aret (arrest)**	-2,5	– Regulation of alternative splicing to enable myofibril maturation in flight muscle (similar to the vertebrate heart)	Spletter et al., 2015
**Odc1 (ornithine decarboxylase 1)**	-1,52	– Targeted overexpression of odc in mice enhances β-adrenergic agonist-induced cardiac hypertrophy	Shantz et al., 2001
**Odc2 (ornithine decarboxylase 1)**	-1,57		
**stv (starvin)**	-1,77	– Tissue-Specific BAG-Domain Protein Required for Larval Food Uptake<br>– Attenuation of starvin leads to Z-band breaks and an irregular sarcomeric actin pattern associated with clustering of myonuclei	Coulson et al., 2005; Wójtowicz et al., 2015

Suppl. Fig. 5: Differentially expressed genes from hearts of H3S28S flies known to be involved in survival/aging/stress resistance and cardiac contractility/heart rate. FC: fold change.

Gene symbol/name	FC
CG6459 protein<br>P32<br>ortholog	+ 1,3
Mitochondrial ribosomal protein L3<br>mRpL3<br>ortholog	+ 1.31
LP10852p<br>mRpS18B<br>ortholog	+ 1,38
Uncharacterized protein, isoform B<br>CG31075<br>ortholog	+ 1.31
Elongation factor Ts, mitochondrial<br>CG6412<br>ortholog	+ 1,32
39S ribosomal protein L19, mitochondrial<br>mRpL19<br>ortholog	+ 1,33
39S ribosomal protein L34, mitochondrial<br>mRpL34<br>ortholog	+ 1,31
Mitochondrial ribosomal protein S31<br>mRpS31<br>ortholog	+ 1,31
Mitochondrial ribosomal protein L42, isoform A<br>mRpL42<br>ortholog	+ 1,45
Probable 28S ribosomal protein S16, mitochondrial<br>mRpS16<br>ortholog	+ 1,34
CG7433, isoform A<br>CG7433<br>ortholog	+ 1,40
CG8043<br>CG8043<br>ortholog	+ 1,40
Mitochondrial ribosomal protein L44<br>mRpL44<br>ortholog	+ 1,33
39S ribosomal protein L33, mitochondrial<br>mRpL33<br>ortholog	+ 1,32

Suppl. Fig. 6: Differentially expressed genes from hearts of H3S28S flies known to be located in the mitochondrial department. FC: fold change.

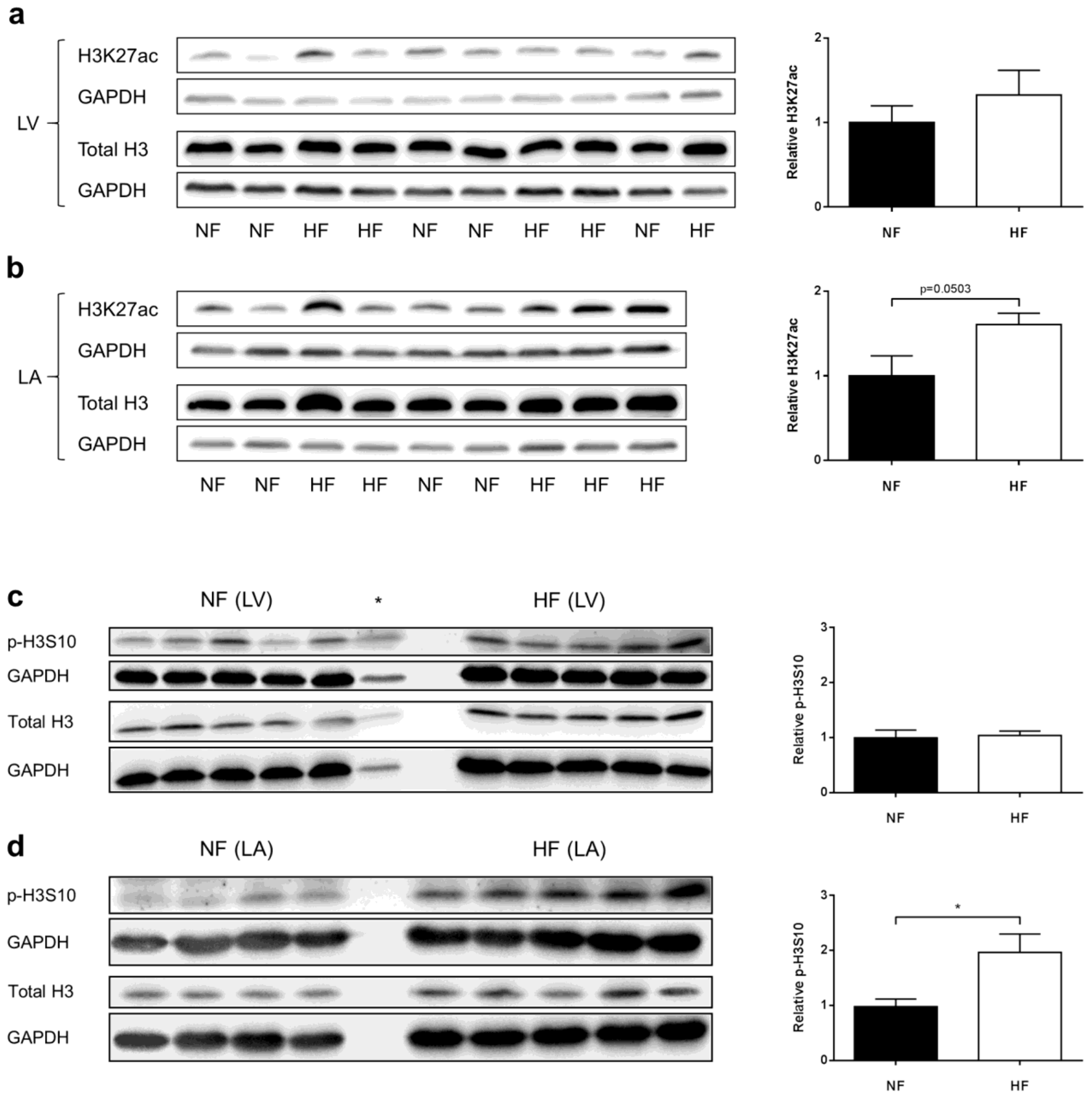

Suppl. Fig. 7: Western Blot analysis of Ac-H3K27 (**a,b**) and p-H3S10 (**c,d**) levels (normalized to total histone H3) in left ventricular (LV; **a,c**) and left atrial (LA; **b,d**) heart tissue of dogs with pacing-induced heart failure (HF) and respective non-failing controls (NF). Asterisk marks NF sample which was excluded from analysis due to protein degradation. All samples were normalized to GAPDH.

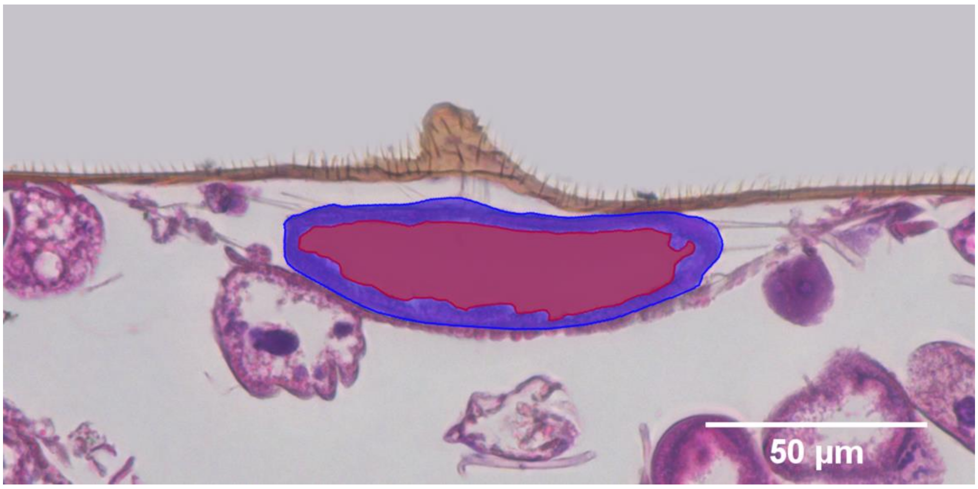

Suppl. Fig. 8: Quantification of heart muscle area (blue) and luminal area (red). Parameters were measured in two serial cross sections (magnification of 40x) and results were averaged.

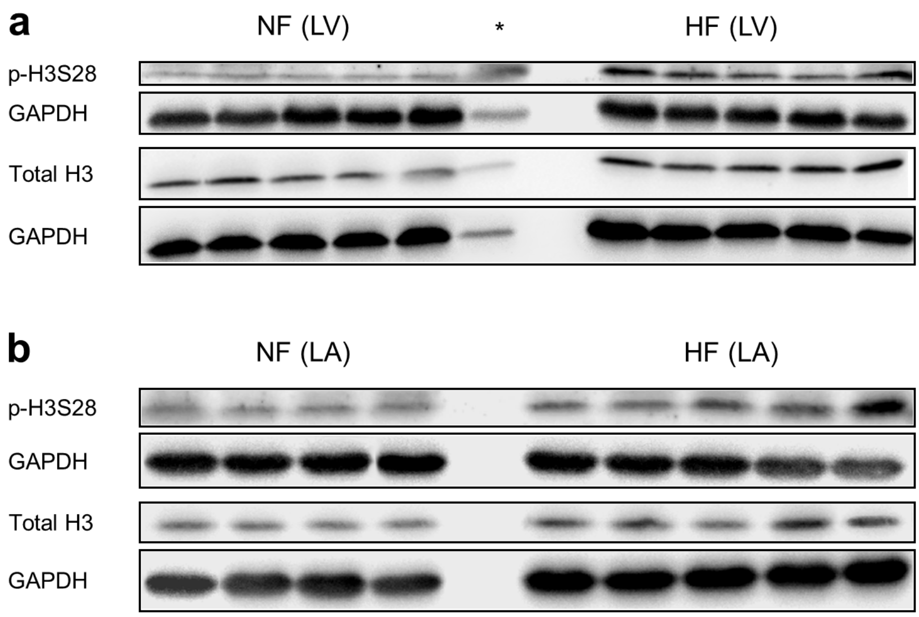

Suppl. Fig. 9: Original blots from Fig. 4. Asterisk marks NF sample which was excluded from analysis due to protein degradation.

Supplementary Material and Methods

OCT measurement of heart rate and fractional shortening

Cardiac function of two day old male flies was measured using a custom built OCT system (Clinical Sensoring and Monitoring, Technische Universitaet Dresden, [1]). Animals were first anesthetized by exposure to Fly Nap® (Carolina Biological Supply Company) and then, with the dorsal side facing the OCT probe, immobilized on a plastic petri dish using double-sided adhesive tape. The conical chamber as the widest part of the heart tube was then imaged for five seconds in transverse direction. After data acquisition at RT, the stage was heated to 37 °C for at least five minutes prior to the second recording after thermal stimulation. Two-dimensional B-mode images were analyzed using custom made, LabVIEW-based software in order to determine heart rate, fractional shortening and the arrhythmicity (AI) index. Heart rate was detected between the first and the last beat within the recording time, fractional shortening was calculated in the horizontal axis as ((EDD-ESD)/EDD)*100. The arrhythmicity index was calculated as the heart period standard deviation normalized to the median heart period. The FD-OCT (fourier-domain) system had a center wavelength of 880 nm and a bandwidth of 130 nm at FWHM (full width at half maximum); an axial and transversal resolution of around 6 µm and 7 µm, respectively, in tissue; an A-scan rate of 12 kHz. For each measurement, around 512 frames (each covering an area of 0.38 × 3 mm², corresponding to 96 × 1024 pixels in the Y−Z direction) were obtained at 93 fps.

Differential gene expression analysis

Dissection of cardiac tubes: The cardiac tubes of seven day old male flies were dissected and exposed according to Vogler and Ocorr [2]. After exposure, cardiac tubes were grasped with forceps at the conical chamber and quickly transferred to an Eppendorf tube containing 350 µl of lysis buffer (Qiagen buffer RLT + 143.35 mM 2-Mercaptoethanol) on ice. The hearts continued to beat immediately following their removal. Until RNA isolation, the Eppendorf tubes containing each 15 cardiac tubes were stored at -80 °C. RNA isolation: Total RNA extraction and DNAse I digestion were carried out using the RNeasy Mini Kit (Qiagen) according to the manufacturer's instructions. Following elution in 30 µl of nuclease free water, RNA was stored at -80 °C. RNA integrity: RNA integrity was evaluated using an RNA 6000 Pico assay on an Agilent 2000 Bioanalyzer according to the manufacturer's instructions. Since the 28S rRNA of most insects contains an endogenous "hidden break", which is disrupted upon heat-denaturation and thereby makes it impossible to calculate a reliable RNA integrity number [3], RNA quality was determined by visual inspection of the electropherogram. RNA sequencing: Complete cDNA was synthesized from 10 ng of mRNA with SmartScribe reverse transcriptase using a universally tailed poly-dT primer and template switching oligo (SMARTer® Ultra™ Low Input RNA Kit for Sequencing - HV, Clontech). This was followed

by amplification of the purified cDNA with the Advantage 2 DNA Polymerase (15 cylces). After ultrasonic shearing of the amplified cDNA (Covaris LE220) samples were subjected to standard Illumina fragment library preparation using the NEBnext chemistries (New England Biolabs), in brief: fragments were end repaired, A-tailed and ligated to indexed Illumina Truseq adapters. Subsequently the NGS libraries were finalized by a universally primed PCR amplification doing 15 cycles. Libraries were purified using XP beads (Beckman Coulter), quantified by qPCR (KAPA Biosysytems) and subjected to Illumina 75 bp single end sequencing on the Illumina HiSeq 2500 (v3 chemistry) platform providing on average 38 Mio reads per sample. Statistical quality control was carried out using RNA-SeQC [4], differential gene expression analysis was performed using DESeq2 [5]. Dmel R6.12 (FB2016_04) served as reference genome/ annotation [6].

Mitochondrial function:

Mitochondrial function analysis was performed as described before [7] and as detailed below

Isolation of mitochondria: 7- to 10-day-old male flies were anesthetized for 5 min at -20 °C. Twenty flies were collected and transferred to 1 ml of isolation buffer (pH 7.4) containing 250 mM sucrose, 10 mM HEPES, 1 mM EGTA with 0.5 % (w/v) bovine serum albumin (BSA). Flies were homogenized using a Teflon pestle. The homogenate was centrifuged at 300 × g for 5 min and the supernatant containing the mitochondria was collected. The supernatant was centrifuged at 6,000 × g for 10 min. The pellet was washed with isolation buffer without BSA and centrifuged at 6,000 × g for 10 min. The pellet was re-suspended in isolation buffer and protein concentration was determined using the LOWRY assay (Biorad). All procedures were performed at 4 °C. Mitochondrial respiration was measured as described before [8,9]. In detail we used a Clark-type electrode (Strathkelvin) at 37 °C during magnetic stirring in respiration buffer containing 125 mM KCl, 10 mM MOPS, 2 mM $MgCl_2$, 5 mM KH_2PO_4, 0.02 mM EGTA, with 5 mM glutamate and 5 mM malate as substrates for complex I. The oxygen electrode was calibrated using a solubility coefficient of 217 nmol O_2/ml at 37 °C. For the measurement of complex I respiration, suspended mitochondria (corresponding to a protein amount of 50 µg) were added to 500 µl of incubation buffer. After 2 min, 1 mM ADP was added and ADP-stimulated respiration was measured for 3 min.

Hereafter, mitochondria were used to either measure maximal uncoupled oxygen uptake in the respiration chamber, or respiration buffer containing mitochondria was taken from the respiration chamber to measure ATP production or ROS production, respectively. Maximal uncoupled respiration was measured by adding 30 nM FCCP (Carbonyl cyanide-4-(trifluoromethoxy)phenylhydrazone) and was analyzed for 30 sec. Respiration was calculated as nmol O_2/min/mg protein using the Stratkelvin analysis software. For every sample, two measurements were performed, results were averaged and

normalized to maximal uncoupled respiration. Mitochondrial ATP production: After measurement of ADP-stimulated respiration, the incubation buffer containing mitochondria was taken from the respiration chamber and immediately supplemented with ATP assay mix (diluted 1:5). ATP production was determined immediately and compared with ATP standards using a 96-well white plate and a Cary Eclipse spectrophotometer (Varian) at 560 nm emission wavelength. Mitochondrial ROS production: ROS concentration in the extramitochondrial space was determined using the Amplex Red Hydrogen Peroxide Assay (Life Technologies). Amplex Red reacts in a 1:1 stoichiometry with peroxides under catalysis by horseradish peroxidase (HRP) and produces highly fluorescent resorufin. The incubation buffer containing mitochondria was removed from the respiration chamber and immediately supplemented with 50 µM Amplex UltraRed and 2 U/ml HRP. The supernatant was collected after 120 min of incubation in the dark. ROS concentration was determined and compared with H_2O_2 standards using a 96-well black plate and a Cary Eclipse fluorescence spectrophotometer (Varian) at 540 nm emission and 580 nm extinction wavelengths.

Supplementary References

1 Schnabel, C., Jannasch, A., Faak, S., Waldow, T. & Koch, E. Ex vivo 4D visualization of aortic valve dynamics in a murine model with optical coherence tomography. *Biomed Opt Express* **5**, 4201-4212, doi:10.1364/BOE.5.004201 (2014).

2 Vogler, G. & Ocorr, K. Visualizing the beating heart in Drosophila. *J Vis Exp*, doi:10.3791/1425 (2009).

3 Winnebeck, E. C., Millar, C. D. & Warman, G. R. Why does insect RNA look degraded? *J Insect Sci* **10**, 159, doi:10.1673/031.010.14119 (2010).

4 DeLuca, D. S. *et al.* RNA-SeQC: RNA-seq metrics for quality control and process optimization. *Bioinformatics* **28**, 1530-1532, doi:10.1093/bioinformatics/bts196 (2012).

5 Love, M. I., Huber, W. & Anders, S. Moderated estimation of fold change and dispersion for RNA-seq data with DESeq2. *Genome Biol* **15**, 550, doi:10.1186/s13059-014-0550-8 (2014).

6 Hoskins, R. A. *et al.* The Release 6 reference sequence of the Drosophila melanogaster genome. *Genome Res* **25**, 445-458, doi:10.1101/gr.185579.114 (2015).

7 Villa-Cuesta, E. & Rand, D. M. Preparation of Mitochondrial Enriched Fractions for Metabolic Analysis in Drosophila. *J Vis Exp*, doi:10.3791/53149 (2015).

8 Gedik, N. *et al.* Cardiomyocyte mitochondria as targets of humoral factors released by remote ischemic preconditioning. *Arch Med Sci* **13**, 448-458, doi:10.5114/aoms.2016.61789 (2017).

9 Kleinbongard, P. *et al.* Pleiotropic, heart rate-independent cardioprotection by ivabradine. *Br J Pharmacol* **172**, 4380-4390, doi:10.1111/bph.13220 (2015).
